# Ecological and Morphological Differentiation Among COI Haplotype Groups in the Plant Parasitic Nematode Species *Mesocriconema Xenoplax*

**DOI:** 10.2478/jofnem-2022-0009

**Published:** 2022-05-11

**Authors:** Julianne N. Matczyszyn, Timothy Harris, Kirsten Powers, Sydney E. Everhart, Thomas O. Powers

**Affiliations:** 1 Department of Plant Pathology, University of Nebraska-Lincoln, Lincoln, NE, 68583-0722; 2 Department of Plant Science and Landscape Architecture, University of Connecticut, Storrs, CT 06269-4067

**Keywords:** parthenogenetic nematodes, population structure, spatial genetic structure, species delimitation, systematics, taxonomy, terrestrial nematodes

## Abstract

DNA barcoding with the mitochondrial COI gene reveals distinct haplotype subgroups within the monophyletic and parthenogenetic nematode species, *Mesocriconema xenoplax*. Biological attributes of these haplotype groups (HG) have not been explored. An analysis of *M. xenoplax* from 40 North American sites representing both native plant communities and agroecosystems was conducted to identify possible subgroup associations with ecological, physiological, or geographic factors. A dataset of 132 *M. xenoplax* specimens was used to generate sequences of a 712 bp region of the cytochrome oxidase subunit I gene. Maximum-likelihood and Bayesian phylogenies recognized seven COI HG (≥99/0.99 posterior probability/bootstrap value). Species delimitation metrics largely supported the genetic integrity of the HG. Discriminant function analysis of HG morphological traits identified stylet length, total body length, and stylet knob width as the strongest distinguishing features among the seven groups, with stylet length as the strongest single distinguishing morphological feature. Multivariate analysis identified land cover, ecoregion, and maximum temperature as predictors of 53.6% of the total variation (*P* = 0.001). Within land cover, HG categorized under “herbaceous,” “woody wetlands,” and “deciduous forest” were distinct in DAPC and RDA analyses and were significantly different (analysis of molecular variance *P* = 0.001). These results provide empirical evidence for molecular, morphological, and ecological differentiation associated with HG within the monophyletic clade that represents the species *Mesocriconema xenoplax*.

DNA barcoding with the COI mitochondrial gene has been widely applied for species identification and biodiversity assessment (Avó *et al*., 2017; Andújar *et al*., 2018; Curry *et al*., 2018; Ge *et al*., 2021; Martoni *et al.*, 2021). Both applications require a broader context in which to interpret the nucleotide sequence data. That context is provided by species delimitation methodology, which provides the tools necessary to establish species boundaries (Janssen *et al*., 2017; Olson *et al*., 2017; Kieneke *et al*., 2021).

DNA barcoding of nematodes with the COI mitochondrial gene is increasingly applied in studies of nematode taxonomy (Derycke *et al*., 2010; Prosser *et al*., 2013; Powers *et al*., 2014; Macheriotou *et al*., 2019; Bai *et al*., 2020; Gonçalves *et al*., 2021). In cases where morphological differences are not detectable, distinct intraspecific clades generated in phylogenetic analyses using the COI marker have supported the existence of cryptic species (Derycke *et al*., 2005, 2008; Fonesca *et al*., 2008; Jörger *et al*., 2012; Palomares-Rius *et al*., 2014). Cryptic nematode species have been identified in marine, freshwater, and terrestrial environments. Such species include *Geomonhystera disjuncta* (Derycke *et al*., 2007), *Thoracostoma trachygaster* (Derycke *et al*., 2010), *Tobrilus gracilis* (Ristau *et al*., 2013), *Caenorhabditis* (Félix *et al*., 2014), and *Pristionchus pacificus* (Herrmann *et al*., 2010; Kanzaki *et al*., 2012). Similarly, plant parasitic nematodes have exhibited COI differentiation in numerous genera (Powers *et al*., 2016, 2018; Olson *et al*., 2017; Palomares-Rius *et al*., 2017; Subbotin *et al*., 2017). Given this measurable genetic differentiation within nematode species, it is possible that a corresponding ecological, geographic, or physiological differentiation also exists. We believe that the parthenogenetic, ectoparasitic, root-feeding nematode *Mesocriconema xenoplax* (Raski, 1952) Loof and De Grisse, 1989 is a good candidate for exploring this linkage. A search for this unrecognized component of differentiation should increase our understanding of the species biology and possibly provide insight into the evolutionary forces that led to its differentiation.

*Mesocriconema xenoplax,* when defined solely based on morphology, includes host associations of more than 25 plant families and has a geographic distribution that includes every continent except Antarctica. This wide range of hosts and habitats suggests that this species is either a remarkable generalist in feeding behavior and tolerance to environmental conditions, or that unrecognized physiological subgroups have evolved within *M. xenoplax.* A recent study of multiple criconematid species, including *M. xenoplax*, using the COI marker and nuclear 18S gene, identified genetic support for subdividing *M. xenoplax* into seven haplotype groups (HG), labeled HG 8 to HG 14 (Powers *et al*., 2014). However, no additional information was provided about the nature of the differentiated groups. In this study, we use multivariate analysis of 132 *M. xenoplax* specimens to search for nematode traits associated with the seven HG.

## Materials and Methods

### Sample selection

A large-scale survey of nematode diversity across diverse ecosystems was conducted previously (Neher *et al*., 1995; Powers *et al*., 2016) to determine if ecoregion boundaries that structure plant and animal communities also structure communities of plant parasitic nematodes. Most of these nematode specimens exist within a curated database that has been characterized both morphologically and molecularly (Powers *et al*., 2020). *Mesocriconema xenoplax* specimens selected from this and subsequent collections were analyzed in the current study. Collection protocols are described in Olson *et al*. (2017). Specimens were assigned a nematode identification number (NID), and DNA sequences for most were submitted to GenBank. Images of selected specimens were also deposited in the Barcode of Life Database (www.barcodinglife.org/). The degenerate COI primer sequences used for amplification were COI-F5 (5′–AATWTWGGTGTTGGAACTTCTTGAAC-3′) and COI-R9 (5′–CTTAAAACATAATGRAAATGWGCWACWACATAATAAGTATC-3′) resulting in an amplicon approximately 790 bp in length and an edited 721-bp sequence for analysis once primer sequences were removed.

### Environmental data

Metadata associated with each site were obtained from the United States Department of Agriculture’s Natural Resource Conservation Service geospatial database (USDA-NRCS, 2017) and assembled into a matrix that included the annual average minimum and maximum temperature and precipitation over 40 years (1981 to 2010). These three continuous variables were categorized by grouping them in increments of 5 years. Associated land cover and elevation for each individual nematode were obtained and viewed using ArcMap software (ESRI, 2011). Associated ecoregion name and biome for each site were identified and recorded into the matrix dataset (Ricketts *et al*., 1999). Estimates of annual, monthly, and event-based climatic parameters were obtained from the USDA-NRCS geospatial database, which used ~800-m^2^ (30 arc-seconds) grids and the PRISM analytical model to generate these estimates. The USDA-NRCS database also provided land cover in a classification system that included 16 categories estimated at a 30-m^2^ resolution: deciduous forest, developed/open space, evergreen forest, woody wetlands, cultivated crops, herbaceous, scrub/shrub, mixed forest, and hay/pasture. Ecoregion and major habitat type were obtained from the World Wildlife Fund based on GPS coordinates (Olson *et al*., 2001). Elevation at each site was recorded at the time of soil sampling using a handheld GPS tracking device. Additionally, specific host associations identified at the time of sample collection, when present, were evaluated for each HG.

### Morphological data

Twenty-four standard morphological measurements previously recorded were used in this study of *M. xenoplax* and included: length, number of body annuli, number of annuli from vulva to tail terminus, number of annuli anterior to excretory pore, pharynx length, stylet length, stylet knob width, mid-body width, vulva position from anterior, vulva position as a percentage of body length, vulval body width, body annulus width, number of anastomoses, width of 10 annuli at mid-body, and width of first labial annuli (Table 1) (Geraert, 2010). No males were encountered in sampling. Only adult females were used in the discriminate function analysis.

**Table 1:** Summary of morphological statistics by HG of *Mesocriconema xenoplax* specimens.

	**HG 08**	**HG 09**	**HG 10**	**HG 11**	**HG 12**	**HG 13**	**HG 14**
N	3	13	9	14	12	16	12
R	99 ± 2.6	94 ± 4.3	94 ± 3.9	101 ± 4.7	95 ± 3.4	98 ± 7.2	100 ± 3.9
Rv	7 ± 0.6	7 ± 0.9	6 ± 1.1	7 ± 0.9	7 ± 0.8	7 ± 1.2	7 ± 0.9
Rex	29 ± 2.1	27 ± 1.6	28 ± 1.9	27 ± 1.8	26 ± 1.6	27 ± 17	27 ± 1.9
Body annule width (µm)	6.4 ± 0.7	7.0 ± 1.0	7.3 ± 0.7	6.0 ± 0.7	6.8 ± 0.7	6.1 ± 0.6	7 ± 0.7
Length (µm)	598.2 ± 43.2	634.6 ± 83.2	621.6 ± 50.5	587.2 ± 63.0	604.8 ± 65.9	579.9 ± 49.3	642 ± 44.7
Stylet length (µm)	83.7 ± 2.1	87.6 ± 5.7	89.6 ± 3.8	72.6 ± 3.0	74.9 ± 3.6	73.3 ± 4.9	71 ± 1.4
Stylet knob width (µm)	13.3 ± 0.6	13.5 ± 0.9	13.3 ± 1.1	12.0 ± 0.7	12.3 ± 1.4	11.6 ± 1.3	14 ± 0.6
VUL	554.2 ± 41.3	594.2 ± 79.4	581.8 ± 52.3	549.7 ± 59.7	565.3 ± 61.0	539.4 ± 46.5	597 ± 40.1
MBW	52.7 ± 1.5	51.8 ± 4.8	55.8 ± 9.1	45.6 ± 6.7	45.6 ± 5.1	47.2 ± 5.6	52 ± 3.7
VBW	40.3 ± 2.5	38.0 ± 3.5	40.3 ± 5.9	35.0 ± 4.2	35.3 ± 4.5	36.7 ± 4.5	40 ± 4.6
ESO	145.7 ± 1.2	159.1 ± 13.7	159.6 ± 6.0	136.2 ± 7.0	141.0 ± 6.6	142.4 ± 6.1	146 ± 8.9
V	92.6 ± 0.8	93.6 ± 1.0	93.5 ± 1.2	93.6 ± 1.2	93.5 ± 0.6	93.0 ± 0.7	93 ± 1.2

HG, haplotype group.

### Sequence alignment and basic statistics

Forward and reverse COI sequences were edited using CodonCode Aligner software version 4.2 (CodonCode Corporation, Centerville, MA; codoncode.com/aligner/), with manual adjustment. Sequences were aligned using ClustalW in MEGA6 (Tamura *et al*., 2013) and Geneious 10.1.3 (Kearse *et al*., 2012). DNA sequences were submitted to GenBank, with associated GPS coordinates when available. Individual and group genetic statistics were calculated in DNAsp (Librado and Rozas, 2009) and MEGA, including: number of polymorphic sites, number of parsimony informative sites, haplotype diversity (Hd), average number of nucleotide differences, number of haplotypes, and average number of base pair differences between sequences.

### Datasets used

Three variations of the original dataset were created for use in our analyses. The original dataset included all *M. xenoplax* sequences analyzed. For sequences from locations without GPS coordinates, but with known county of origin, spatial location was imputed using the county centroid from Google Maps (Fresno, CA). The second dataset excluded individuals with a unique gene sequence (singletons) and retained only those with ≥2 individuals per gene sequence. The third dataset excluded individuals collected outside of the United States, due to the limited metadata resources. The fourth dataset excluded individuals that were both genetically and environmentally identical to one another (e.g. clone-corrected dataset), and used in analyses affected by overrepresentation.

### Phylogenetic analysis

Three types of phylogenetic trees were constructed, with *Mesocriconema inaratum* (Hoffman, 1974) Powers et al., 2014 included as an outgroup. The neighbor-joining and maximum-likelihood trees were reconstructed in MEGA, and the Bayesian tree in Geneious. Two evolutionary models were applied within the neighbor-joining tree, Jukes-Cantor (JC69) and Kimura 2-Parameter (K2P). Neighbor-joining and maximum-likelihood trees were run with 2,000-bootstrap replications (Shao and Tu, 2012). Maximum-likelihood and Bayesian analysis used jModelTest for model selection and Akaike information criterion (Akaike, 1998) and Akaike information criterion corrected values (Hurvich and Tsai, 1989). For both maximum-likelihood and Bayesian trees, an HKY+G+I model was the best fit with the lowest Akaike information criterion and Akaike information criterion corrected values. The Bayesian inference tree was supported with 2,000-posterior probabilities rankings, run for two million generations (ngen), a heating scheme (temp = 0.06), sampling each 5,000 generations (sampled frequency), with burn in of 250,000, and remaining samples were used to compute the consensus tree. Convergence was confirmed when the average standard deviation (SD) of split frequencies was less than 0.01.

### Species delimitation

Each species delimitation method utilized the original dataset, with the exception of Birky’s *K/θ* analysis, which censored singletons (i.e., first dataset variation). Cladistic statistics were calculated using the species delimitation plugin within the Geneious software package (Masters *et al*., 2011). The plugin included assessments of overall monophyly and intra- or inter-distance ratio. This ratio, together with the known number of taxa in the reference group, was used to estimate the probability of correct identification under the conservative P ID(Strict) criteria based on variation within as compared to between. The relaxed, P ID(Liberal) criteria is based on a-priori groupings with a cut off for each group at >80% (Ross *et al*., 2008; Hamilton *et al*., 2014). Rosenberg’s P(AB) (Rosenberg, 2007) and Rodrigo’s P(AD) (Rodrigo *et al*., 2008) measured the probability that the observed patterns identifying reciprocal monophyly were due to random coalescent processes (Prévot *et al*., 2013).

Statistical parsimony analysis was conducted on individual HG using the program TCS 1.21 (Clement *et al*., 2000) with a 90-connection limit. Automatic Barcode Gap Discovery (ABGD) analysis is an online platform (http://wwwabi.snv.jussieu.fr/public/abgd/) that applies a clustering algorithm to determine the optimal threshold (Puillandre *et al*., 2012). Three models of sequence evolution were applied – JC69, K2P, and simple distance – along with the default settings, consisting of relative gap width (*X* = 1.50) and intraspecific divergence values (*P_min_* < 0.001 and *P_max_* = 0.10), where correct species estimates are projected to correspond to *P* = 0.01. To test for parthenogenetic speciation, the 4× rule, Birky’s *K/θ* > 4, was applied, which utilized the threshold between clades to delimit reciprocal monophyly in asexual parthenogenetic species (Birky *et al*., 2005). The estimator for interclade divergence *K* was calculated as observed average base pair distance between clades corrected for multiple hits. The intraclade variation estimator *θ* is calculated as π(1–4π/3), with π representing the relativized nucleotide diversity that is corrected for sample size. Calculations were done in an Excel spreadsheet. The ratio rule indicates that if the ratio *K*/*θ* is greater than four, it can be stated with 95% confidence that these two clades have arisen solely by neutral genetic drift (Birky, 2013).

### Phenotypic variation

Under the assumption that two HG can diverge morphologically over time, the association of genetic structure was compared with the morphological variation described above, using a discriminant function analysis conducted in JMP®, Version 7 (SAS Institute Inc., Cary, NC, 1989–2007). The discriminant function analysis uses an algorithm that classifies cases into previously determined groups and derives a model that best discriminates groups, maximizing intragroup variation relative to inter-group variation (Friedman, 1989; McClure *et al*., 2003; Härdle and Simar, 2007). Prior to analysis, juvenile specimens, singletons, and specimens lacking all morphological measurements were omitted. A quadratic discriminant function was computed using the within-group covariance matrices that fit the data best, and a backward stepwise procedure was used to assess the discriminatory power of variable combinations. Results were interpreted by a canonical plot with ellipses representing the confidence intervals of groups determined a priori. The amount of overlap between the ellipses correlates with the degree to which there is morphological distinction or separation between the groups. If there was no overlap, then there was significant difference between the two groups morphologically. The canonical plot also contains bi-plot rays that indicate which ones, among the variables tested, were the strongest predictors of the data, as indicated by the length of the ray and the direction the ray was pointing to with respect to the other plotted rays.

### Spatial genetic structure

The association between geographical distance within and between HG and the intra-genetic distance of the respective HG was assessed using the Mantel test (Mantel, 1967) in R software version 1.1.383 (R Core Team, 2018) using the mantel() function in the R package “ecodist” 2.0.1 (Goslee and Urban, 2007). This analysis assumes that a consequence of dispersal limited by geographic distance, isolation by distance, is correlated (positively or negatively) with the organisms’ genetic distance. This relationship can be positive, genetically similar, and geographically close together, or genetically dissimilar and geographically far apart. It can also be negative and genetically similar, but geographically far apart, or genetically dissimilar but geographically close together. The detection of a correlation indicates an effect of dispersal and/or migration probabilities within the individual HG. A pairwise distance matrix of genetic similarity between individual sequences within each HG was created using the JC69 substitution model on the dataset without singletons and without nematodes collected outside the United States. The JC69 assumes equal base pair frequencies and equal mutation rates. Geographic distance between individuals was also calculated in a pairwise fashion, using latitudinal and longitudinal coordinates and calculated using the geodesic() function in the R package “geosphere” 1.5–7 (Hijmans, 2017). This function calculates the shortest distance between two points following an ellipsoid (Hijmans, 2015). To determine whether there was increasing genetic similarity with decreasing distance, the two matrices were tested for structure along a spatial gradient with 10,000 permutations, 500-bootstrap iterations, a 0.90-resampling level, and a Pearson correlation coefficient with a 0.95-confidence level. The power and false-positive rates were estimated based on the significance threshold value of α = 0.05, the Mantel coefficient, MantleR (*r*) with a two-tailed test, and a null hypothesis *r* = 0. The Mantel coefficient was calculated for each HG individually, with *P*-value, and lower and upper limits.

### Population structure

To assess for associations between population structure and environmental parameters, a distance-based redundancy analysis (dbRDA) was applied (Legendre and Anderson, 1999). This analysis does not require groups to be defined a priori and relies on a forward–backward selection process to identify the model of environmental parameters that best predicts the genetic variation. This method detects linear relationships based on similarities and dissimilarities, generated by constrained ordination on a distance matrix representing the response variable, to describe the relative contribution of multiple independent explanatory variables (McClure *et al*., 2013; Kamvar *et al*., 2017). The analysis was first performed using the censored singletons (i.e., first dataset variation), and then using the clone-corrected dataset (i.e., third dataset variation). Input data for the dbRDA included a pairwise genetic distance matrix that was calculated using the JC69 model and environmental variables corresponding to each individual, supplied as a data frame. The dbRDA was computed in R using the package “vegan” 2.4–3 (Oksanen *et al*., 2013), and the function capscale() was used to eliminate collinear environmental variables with a correlation ≥0.75. Among correlated environmental variables, the variable that described the most genetic variation was retained in further analysis. A forward–backward selection process was applied with the “vegan” function ordistep() to identify the model that best described the genetic variability between HG using an automatic stepwise building and selection method on each possible model combination via permutation tests (Oksanen, 2015). An analysis of variance was performed to test for the significance of the reduced model and marginal effects using 999 permutations. The environmental variables with the strongest associations were selected for further analysis.

The strongest association was assigned by the visualization within the bi-plots. Principal components are indicated by an axis, with each variable or HG indicated a priori and projected onto the axis (Jongman, 1995). The strength of a variable in association with the HG’s genetic score is conveyed by the length of the bi-plot rays and the relative distance of the ray in relation to the HG’s location on the bi-plot figure. This plot indicates the presence of a correlation between HG and the bi-plot axes. The eigenvalues for these axes also indicate the importance of the subsequent variables associated with the axes, as well as other HG, in order to explain the relationship within the data matrices (Fiscus and Neher, 2002).

A discriminant analysis of principal components was implemented using the R package “adegenet” 2.1.0 to assess the per-sample posterior group assignment probability (Jombart *et al*., 2010). Clone-corrected environmental and sequence data (i.e., third dataset variation) were used in this analysis, in addition to censoring groups with fewer than five individuals per variable category. This analysis first analyzes the transformed data in a principal component analysis, followed by an analysis of the resulting principal components in a linear discriminant analysis to optimize variation between HG while minimizing variation within each HG (Jombart *et al*., 2010, Jombart and Collins, 2015). The optimal number of principal components was assessed and selected by the cross-validation function xvalDapc(). This function implements a procedure to iterate over an increasing number of principal components on a subset (90%) of the data, while maximizing the lowest mean squared error. Percent reassignment of each individual to its corresponding land cover was visualized using stacked bar plots in R.

HG were defined according to the strongest environmental variables, and those containing fewer than five individuals were compared using an analysis of molecular variance (AMOVA) in the R package “pegas” version 0.10 (Excoffier *et al*., 1992; Paradis, 2010). The AMOVA utilized a JC69 pairwise genetic distance matrix and evaluated population differentiation within and between HG, estimated with the phi statistic (*ϕ*). Significant differences were determined using the function randtest() from R package “ade4” version 1.7–10, using 9,999-bootstrap replicates (Dray and Dufour, 2007). For environmental variables that resulted in more than two HG being defined, pairwise comparison between individuals within a HG were made using AMOVA to determine which HG were significantly different.

## Results

A total of 132 specimens were identified as *M. xenoplax* and selected for the present study, representing 40 sites within 16 states and 14 ecoregions, with 33 specimens associated with agroecosystems (Table 2). Of the 132 individuals, 92 were adult females, 31 were juvenile, and the developmental stage was unidentifiable for 9 specimens. There were 54 unique haplotypes among the 132 *M. xenoplax* COI sequences.

**Table 2:** Locality and GenBank accession information for *Mesocriconema xenoplax* specimens in this study.

**NID**	**Locality**	**Ecoregion**	**Agricultural site (Y or N)**	**GenBank Accession No.**
N3	Timmas Farm Ecological Forest Reserve, Cass County, NE, USA	Central Tall Grasslands	N	KJ787901
N173	Middle Loup River, Hooker County, NE, USA	Nebraska Sand Hills Mixed Grasslands	N	KJ787902
N583	Wakulla Springs State Park, Wakulla County, FL, USA	Southeastern Conifer Forests	N	KJ787880
N584	Ichetucknee Springs State Park, Columbia County, FL, USA	Southeastern Conifer Forests	N	KJ787885
N588	Ichetucknee Springs State Park, Columbia County, FL, USA	Southeastern Conifer Forests	N	KJ787886
N607	Schluckebier Prairie State Natural Area, Sauk County, WI, USA	Upper Midwest forest-savanna Transition Zone	N	KJ787881
N724	Chimney Creek, Great Smoky Mountains National Park, USA	Appalachian Blue Ridge Forests	N	KJ787906
N728	Pickens County, SC, USA	Southeastern Mixed Forests	Y	KJ787873
N729	Pickens County, SC, USA	Southeastern Mixed Forests	Y	KJ787874
N730	Pickens County, SC, USA	Southeastern Mixed Forests	Y	KJ787875
N733	Ozark National Forest, AR, USA	Central US Hardwood Forests	N	KJ787882
N735	Ozark National Forest, AR, USA	Central US Hardwood Forests	N	KJ787883
N736	Southeastern Fruit and Nut Research Station, Peach County, GA, USA	Southeastern Mixed Forests	Y	KJ787887
N746	Crawford Bay, British Columbia, Canada	Cascade Mountains Leeward Forests	N	KJ787907
N747	Crawford Bay, British Columbia, Canada	Cascade Mountains Leeward Forests	N	KJ787908
N944	Timmas Farm Ecological Forest Reserve, Cass County, NE, USA	Central Tall Grasslands	N	KJ787903
N945	Timmas Farm Ecological Forest Reserve, Cass County, NE, USA	Central Tall Grasslands	N	KJ787904
N947	Timmas Farm Ecological Forest Reserve, Cass County, NE, USA	Central Tall Grasslands	N	KJ787905
N999	Albright Grove, Great Smoky Mountains National Park, USA	Appalachian Blue Ridge Forests	N	KJ787896
N1024	Albright Grove, Great Smoky Mountains National Park, USA	Appalachian Blue Ridge Forests	N	KJ787897
N1025	Albright Grove, Great Smoky Mountains National Park, USA	Appalachian Blue Ridge Forests	N	KJ787898
N1028	Albright Grove, Great Smoky Mountains National Park, USA	Appalachian Blue Ridge Forests	N	KJ787899
N1072	Nine-Mile Prairie, Lancaster County, NE, USA	Central Tall Grasslands	N	KJ787913
N1073	Nine-Mile Prairie, Lancaster County, NE, USA	Central Tall Grasslands	N	KJ787914
N1215	Long Branch Stream Valley Park, Fairfax County, VA, USA	Southeastern Mixed Forests	N	KJ787900
N1216	Long Branch Stream Valley Park, Fairfax County, VA, USA	Southeastern Mixed Forests	N	MN711175
N1217	Spring Green Prairie Preserve, Sauk County, WI, USA	Upper Midwest forest-savanna Transition Zone	N	KJ787884
N1267	George Washington Memorial Parkway, Fairfax County, VA, USA	Southeastern Mixed Forests	N	KJ787909
N1276	George Washington Memorial Parkway, Fairfax County, VA, USA	Southeastern Mixed Forests	N	KJ787910
N1294	Musser Fruit Research Farm, Oconee County, SC, USA	Southeastern Mixed Forests	Y	KJ787876
N1297	Musser Fruit Research Farm, Oconee County, SC, USA	Southeastern Mixed Forests	Y	KJ787877
N1298	Musser Fruit Research Farm, Oconee County, SC, USA	Southeastern Mixed Forests	Y	KJ787878
N1327	Fresno County, CA, USA	California Central Valley	Y	KU236636
N1346	George Washington Memorial Parkway, Fairfax County, VA, USA	Southeastern Mixed Forests	N	KY574831
N1361	George Washington Memorial Parkway, Fairfax County, VA, USA	Southeastern Mixed Forests	N	KY574643
N1368	Nine-Mile Prairie, Lancaster County, NE, USA	Central tall grasslands	N	KJ787915
N1375	Nine-Mile Prairie, Lancaster County, NE, USA	Central tall grasslands	N	KJ787916
N1397	George Washington Memorial Parkway, VA, USA	Southeastern Mixed Forests	N	KY574644
N1451	George Washington Memorial Parkway, VA, USA	Southeastern Mixed Forests	N	KJ788063
N2262	George Washington Memorial Parkway, VA, USA	Southeastern Mixed Forests	N	KY574645
N2269	George Washington Memorial Parkway, VA, USA	Southeastern Mixed Forests	N	MN711176
N2528	Nine-Mile Prairie, Lancaster County, NE, USA	Central tall grasslands	N	KY574650
N2557	Autauga County, AL, USA	Southeastern Mixed Forests	N	KY574633
N2558	Autauga County, AL, USA	Southeastern Mixed Forests	Y	MN711177
N2577	Chilton County, AL, USA	Southeastern Mixed Forests	Y	KY574624
N2604	Chilton County, AL, USA	Southeastern Mixed Forests	Y	MN711178
N2610	Chilton County, AL, USA	Southeastern Mixed Forests	Y	MN711179
N2611	Chilton County, AL, USA	Southeastern Mixed Forests	Y	MN711180
N2615	Chilton County, AL, USA	Southeastern Mixed Forests	Y	KY574634
N2618	Chilton County, AL, USA	Southeastern Mixed Forests	Y	KY574635
N2619	Chilton County, AL, USA	Southeastern Mixed Forests	Y	MN711181
N2622	Chilton County, AL, USA	Southeastern Mixed Forests	Y	KY574623
N2694	Albright Grove, Great Smoky Mountains National Park, USA	Appalachian Blue Ridge Forests	N	MF770909
N2719	Albright Grove, Great Smoky Mountains National Park, USA	Appalachian Blue Ridge Forests	N	MN711182
N2720	Albright Grove, Great Smoky Mountains National Park, USA	Appalachian Blue Ridge Forests	N	MN711183
N2727	Purchase Knob, Great Smoky Mountains National Park, USA	Appalachian Blue Ridge Forests	N	MN711184
N2728	Purchase Knob, Great Smoky Mountains National Park, USA	Appalachian Blue Ridge Forests	N	MN711185
N2842	Double Springs Gap, Great Smoky Mountains National Park, USA	Appalachian Blue Ridge Forests	N	MN711186
N2844	Double Springs Gap, Great Smoky Mountains National Park, USA	Appalachian Blue Ridge Forests	N	MN711187
N2849	Konza Prairie Biological Station, Riley County, KS, USA	Flint Hills Tall Grasslands	N	KY574651
N2850	Konza Prairie Biological Station, Riley County, KS, USA	Flint Hills Tall Grasslands	N	KY574652
N2851	Konza Prairie Biological Station, Riley County, KS, USA	Flint Hills Tall Grasslands	N	MN711188
N2853	Konza Prairie Biological Station, Riley County, KS, USA	Flint Hills Tall Grasslands	N	MN711189
N2855	Double Springs Gap, Great Smoky Mountains National Park, USA	Appalachian Blue Ridge Forests	N	MN711190
N2857	Double Springs Gap, Great Smoky Mountains National Park, USA	Appalachian Blue Ridge Forests	N	MN711191
N2858	Double Springs Gap, Great Smoky Mountains National Park, USA	Appalachian Blue Ridge Forests	N	MN711192
N2863	Konza Prairie Biological Station, Riley County, KS, USA	Flint Hills Tall Grasslands	N	KY574653
N2864	Konza Prairie Biological Station, Riley County, KS, USA	Flint Hills Tall Grasslands	N	MN711193
N2869	Double Springs Gap, Great Smoky Mountains National Park, USA	Appalachian Blue Ridge Forests	N	KY574646
N2873	Double Springs Gap, Great Smoky Mountains National Park, USA	Appalachian Blue Ridge Forests	N	MN711194
N2874	Double Springs Gap, Great Smoky Mountains National Park, USA	Appalachian Blue Ridge Forests	N	MN711195
N2891	Konza Prairie Biological Station, Riley County, KS, USA	Flint Hills Tall Grasslands	N	KY574654
N2892	Konza Prairie Biological Station, Riley County, KS, USA	Flint Hills Tall Grasslands	N	KY574655
N2893	Konza Prairie Biological Station, Riley County, KS, USA	Flint Hills Tall Grasslands	N	MN711196
N2896	Konza Prairie Biological Station, Riley County, KS, USA	Flint Hills Tall Grasslands	N	MN711197
N2933	West Point, Great Smoky Mountains National Park, USA	Appalachian Blue Ridge Forests	N	KY574647
N2945	West Point, Great Smoky Mountains National Park, USA	Appalachian Blue Ridge Forests	N	MN711198
N2998	Cades Cove, Great Smoky Mountains National Park, USA	Appalachian Blue Ridge Forests	N	MN711199
N3008	Goshen Prong, Great Smoky Mountains National Park, USA	Appalachian Blue Ridge Forests	N	KY574648
N3013	Trillium Gap, Great Smoky Mountains National Park, USA	Appalachian Blue Ridge Forests	N	MN711200
N3078	Gifford Woods State Park, VT, USA	New England Acadian Forests	N	KY574639
N3084	Hayden Prairie State Preserve, Howard County, IA, USA	Central tall grasslands	N	MN711201
N3112	Hayden Prairie State Preserve, Howard County, IA, USA	Central tall grasslands	N	MN711202
N3124	Hayden Prairie State Preserve, Howard County, IA, USA	Central tall grasslands	N	KY574656
N3153	George Washington Memorial Parkway, VA, USA	Southeastern Mixed Forests	N	KY574649
N3154	George Washington Memorial Parkway, VA, USA	Southeastern Mixed Forests	N	MN711203
N3208	Ichetucknee Springs State Park, FL, USA	Southeastern Conifer forests	N	KY574625
N3246	Oconaluftee, Great Smoky Mountains National Park, USA	Appalachian Blue Ridge Forests	N	MN711204
N3253	Oconaluftee, Great Smoky Mountains National Park, USA	Appalachian Blue Ridge Forests	N	MN711205
N3316	Twin Creeks, Great Smoky Mountains National Park, USA	Appalachian Blue Ridge Forests	N	KY574640
N3320	Twin Creeks, Great Smoky Mountains National Park, USA	Appalachian Blue Ridge Forests	N	KY574832
N3342	Torreya State Park, FL, USA	Southeastern Conifer forests	N	KY574636
N3361	Raspberry Island, Apostle Islands National Lakeshore, WI, USA	Western Great Lakes Forest	N	KY574641
N3371	Raspberry Island, Apostle Islands National Lakeshore, WI, USA	Western Great Lakes Forest	N	MN711206
N3372	Raspberry Island, Apostle Islands National Lakeshore, WI, USA	Western Great Lakes Forest	N	MN711207
N3373	Raspberry Island, Apostle Islands National Lakeshore, WI, USA	Western Great Lakes Forest	N	MN711208
N3374	Raspberry Island, Apostle Islands National Lakeshore, WI, USA	Western Great Lakes Forest	N	MN711209
N3481	Wakulla Springs State Park, FL, USA	Southeastern Conifer forests	N	MN711210
N3491	West Point, Great Smoky Mountains National Park, USA	Appalachian Blue Ridge Forests	N	MF770951
N3492	West Point, Great Smoky Mountains National Park, USA	Appalachian Blue Ridge Forests	N	MN711211
N5508	Oconaluftee, Great Smoky Mountains National Park, USA	Appalachian-Blue Ridge Forests	N	MN711216
N5587	Tuskegee National Forest, Macon County, AL, USA	Southeastern Mixed Forests	N	KY574626
N5592	Canyonlands-South, Big Thicket National Preserve, TX, USA	Piney Woods forests	N	KY574627
N5593	Canyonlands-South, Big Thicket National Preserve, TX, USA	Piney Woods forests	N	KY574628
N5603	Canyonlands-South, Big Thicket National Preserve, TX, USA	Piney Woods forests	N	MF770959
N5638	Tuskegee National Forest, Macon County, AL, USA	Southeastern Mixed Forests	N	KY574629
N5643	Big Sandy Creek, Big Thicket National Preserve, TX, USA	Piney Woods forests	N	KY574630
N5645	Big Sandy Creek, Big Thicket National Preserve, TX, USA	Piney Woods forests	N	KY574631
N5712	Ichetucknee Springs State Park, FL, USA	Southeastern Conifer forests	N	MN711217
N5713	Ichetucknee Springs State Park, FL, USA	Southeastern Conifer forests	N	MN711218
N5714	Ichetucknee Springs State Park, FL, USA	Southeastern Conifer forests	N	MN711219
N5715	Ichetucknee Springs State Park, FL, USA	Southeastern Conifer forests	N	MN711220
N5726	Black Hills National Forest, Lawrence County, SD, USA	South Central Rockies forests	N	KY574642
N5727	Black Hills National Forest, Lawrence County, SD, USA	South Central Rockies forests	N	MN711221
N5728	Black Hills National Forest, Lawrence County, SD, USA	South Central Rockies forests	N	MN711222
N5731	Black Hills National Forest, Lawrence County, SD, USA	South Central Rockies forests	N	MN711223
N5813	Cuming County, NE, USA	Central Tall Grasslands	Y	KY574637
N5814	Cuming County, NE, USA	Central Tall Grasslands	Y	MN711224
N5815	Cuming County, NE, USA	Central Tall Grasslands	Y	MN711225
N5816	Cuming County, NE, USA	Central Tall Grasslands	Y	KY574632
N5943	Tulare County, CA, USA	Central California Valley	Y	KY574638
P74053	Fresno County, CA, USA	Central California Valley	Y	KJ787911
P194033	Konza Prairie Biological Station, Riley County, KS, USA	Flint Hills Tall Grasslands	Y	KJ787912
P231026	Southeastern Fruit and Nut Research Station, Peach County, GA, USA	Southeastern Plains	Y	KJ787888
P231028	Southeastern Fruit and Nut Research Station, Peach County, GA, USA	Southeastern Plains	Y	KJ787889
P231030	Southeastern Fruit and Nut Research Station, Peach County, GA, USA	Southeastern Plains	Y	KJ787879
P231031	Southeastern Fruit and Nut Research Station, Peach County, GA, USA	Southeastern Plains	Y	KJ787890
P231032	Southeastern Fruit and Nut Research Station, Peach County, GA, USA	Southeastern Plains	Y	KJ787891
P231034	Southeastern Fruit and Nut Research Station, Peach County, GA, USA	Southeastern Plains	Y	KJ787892
P231035	Southeastern Fruit and Nut Research Station, Peach County, GA, USA	Southeastern Plains	Y	KJ787893
P231036	Southeastern Fruit and Nut Research Station, Peach County, GA, USA	Southeastern Plains	Y	KJ787894
P231037	Southeastern Fruit and Nut Research Station, Peach County, GA, USA	Southeastern Plains	Y	KJ787895

NID, nematode identification number.

### Phylogenetic analysis

**Figure 1:**
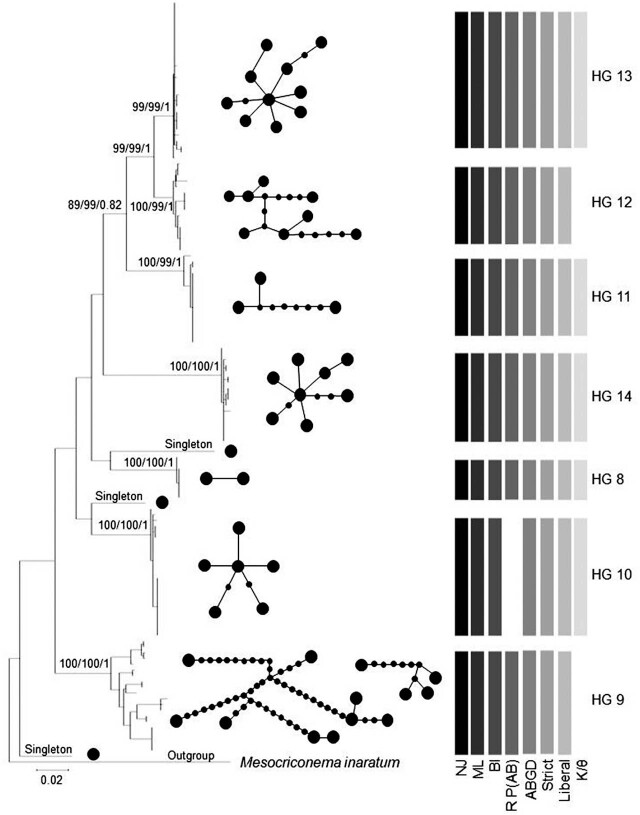
A condensed maximum-likelihood tree of 721-bp sequences isolated from the COI gene for each individual in the morphospecies *Mesocriconema xenoplax*. Generated in the program MEGA, the tree displays seven partitions resulting in the HG label for further analysis. Bootstrap values from neighbor-joining and maximum-likelihood trees and posterior probability from Bayesian trees showing support for each HG are given at the terminal node, neighbor-joining, maximum-likelihood, and Bayesian, respectively. Each analysis is indicated by shading in the legend. The absence of the bar indicates the absence of support. The spanning tree network of each HG constructed using statistical parsimony is given within each HG bracket. Larger circles represent a unique haplotype. Smaller circles represent the number of mutational steps between haplotypes. Networks are disconnected when they exceed the 90% connection limit. HG, haplotype group.

Figure 1 displays a maximum-likelihood tree of the unique COI haplotypes. The bootstrap and posterior probability support values for HG are labeled at defining nodes sequentially for neighbor-joining, maximum-likelihood, and Bayesian analyses. Two more ancestral nodes are labeled to illustrate the close relationship between HG 12 and 13, and group 11 as a sister group to the pair. Each of the seven HG are supported by posterior probability values of 1.0 in Bayesian analyses and bootstrap values of 99 to 100 for neighbor-joining and maximum-likelihood trees.

### Species delimitation

The seven HG supported by phylogenetic analyses served as primary species hypotheses for testing by other species delimitation approaches. HG 8, 11, 13, and 14 had significant support from each of the species delimitation methods (Fig. 1). These four groups were characterized by (i) the statistical parsimony network analysis indicating 90% confidence level resulting in a single interconnected network, (ii) distinct recursive partitioning (*P* = 0.012) for the ABGD analysis, (iii) statistical significance for reciprocal monophyly for *K/θ* (Table 3), and (iv) statistical significance (<0.001) from the Rosenberg’s P(AB), P ID(Liberal), and P ID(Strict) (≤0.90) analyses calculated within the Geneious species delimitation plugin software (Table 4). Statistics calculated for each HG using DNAsp software identified HG 8 as having low genetic variability as indicated by scoring the lowest among the seven HG on seven parameters (Table 5). A significant isolation by distance relationship was identified for HG 8, 9, 10, and 11 (*P* ≤ 0.050; Table 6).

**Table 3:** Calculations of Birky’s 4× test on the morphospecies *Mesocriconema xenoplax* HG interclade divergence (*K*), intraclade variation (*θ*), and estimate of reciprocal monophyly (*K/θ*).

***HG***	***K* ^a^**	***θ* ^b^**	***K*/*θ*^c^**
13	0.029	0.006	4.833^*^
12	0.029	0.031	0.935
11	0.060	0.006	10.000^*^
14	0.102	0.009	11.333^*^
8	0.079	0.003	26.333^*^
10	0.086	0.006	14.333^*^
9	0.089	0.096	0.927

a Interclade divergence calculated as the observed average distance between clades corrected for multiple hits (Birky, 2013).

b Interclade variation calculated as π(1–4π/3), with π equal to the relativized nucleotide diversity that is corrected for sample size (Birky, 2013).

c Degree of monophyly within the group, where values greater than 4.0 indicate reciprocal monophyly.

* 95% confidence of reciprocal monophyly.

HG, haplotype group.

**Table 4:** Species delimitation statistics corresponding to the neighbor-joining tree HG built from the COI gene sequence for the morphospecies *Mesocriconema xenoplax*. Statistics include the intra-distance of each HG, inter-distance of each HG compared to its sister clade, ratio of intra- and inter-distance, P ID(strict), P ID(liberal), Av(MRCA-tips), Rodrigo’s P(AD), and Rosenberg’s P(AB). HG are organized in the table below according to their phylogenetic relationships shown.

**HG**	**Closest HG**	**Intra-distance**	**Inter-distance - closest**	**Intra/inter**	**P ID (Strict)**	**P ID (Liberal)**	**Av(MRCA-tips)**	**P(AD)**	**R(AB)**
13	12	0.004	0.03	0.13	0.95	0.98	0.002	0.24	7.00E−14
12	13	0.007	0.03	0.23	0.92	0.97	0.005	1.00	7.00E−14
11	13	0.002	0.063	0.02	0.99	1.00	0.004	0.05	3.60E−18
14	8	0.008	0.118	0.07	0.97	0.99	0.004	0.05	1.40E−24
8	13	<0.001	0.095	0.01	0.94	1.00	<0.001	0.05	6.70E−15
10	8	0.005	0.098	0.05	0.98	1.00	0.004	0.41	3.62E−03
9	8	0.03	0.104	0.29	0.90	0.97	0.020	0.56	3.90E−26

**Table 5:** Summary of genetic statistics for the 721-bp sequence isolated from the COI gene for each individual HG among the seven HG identified within the morphospecies *Mesocriconema xenoplax*. HG are organized in the table below according to their phylogenetic relationships shown.

**HG**	**n^a^**	**S^b^**	**PI^c^**	**Hd^d^**	**k^e^**	**h^f^**	**Pi^g^**
13	28	11	5	0.738	1.354	10	0.002
12	16	15	9	0.858	4.683	8	0.006
11	16	8	2	0.342	1.308	3	0.002
14	18	10	6	0.863	1.941	8	0.003
8	8	1	1	0.429	0.428	2	<0.001
10	23	7	3	0.711	1.628	6	0.002
9	20	60	50	0.932	19.53	13	0.027
Pooled	132	214	182	0.963	56.15	53	0.078

a Number of individuals.

b Polymorphic sites.

c Parsimony informative sites.

d Hd.

e Average number of nucleotide differences.

f Number of haplotypes.

g Nucleotide diversity.

Hd, haplotype diversity; HG, haplotype group.

**Table 6:** Isolation by distance metric calculated by Mantel test statistic (Mantel R), and associated *P*-value for each of the seven HG within the morphospecies *Mesocriconema xenoplax*. HG are organized in the table below according to their phylogenetic relationships shown.

**HG**	**Mantel R**	***P*-value**
13	0.048	0.731
12	0.206	0.118
11	0.989	<0.001^*^
14	–0.108	0.443
8	0.874	0.021^*^
10	0.627	<0.001^*^
9	0.284	0.005^*^

* Significant Mantel R scores at *P* = 0.05.

HG, haplotype group.

### Geographic distribution of HG 11 to 13

**Figure 2:**
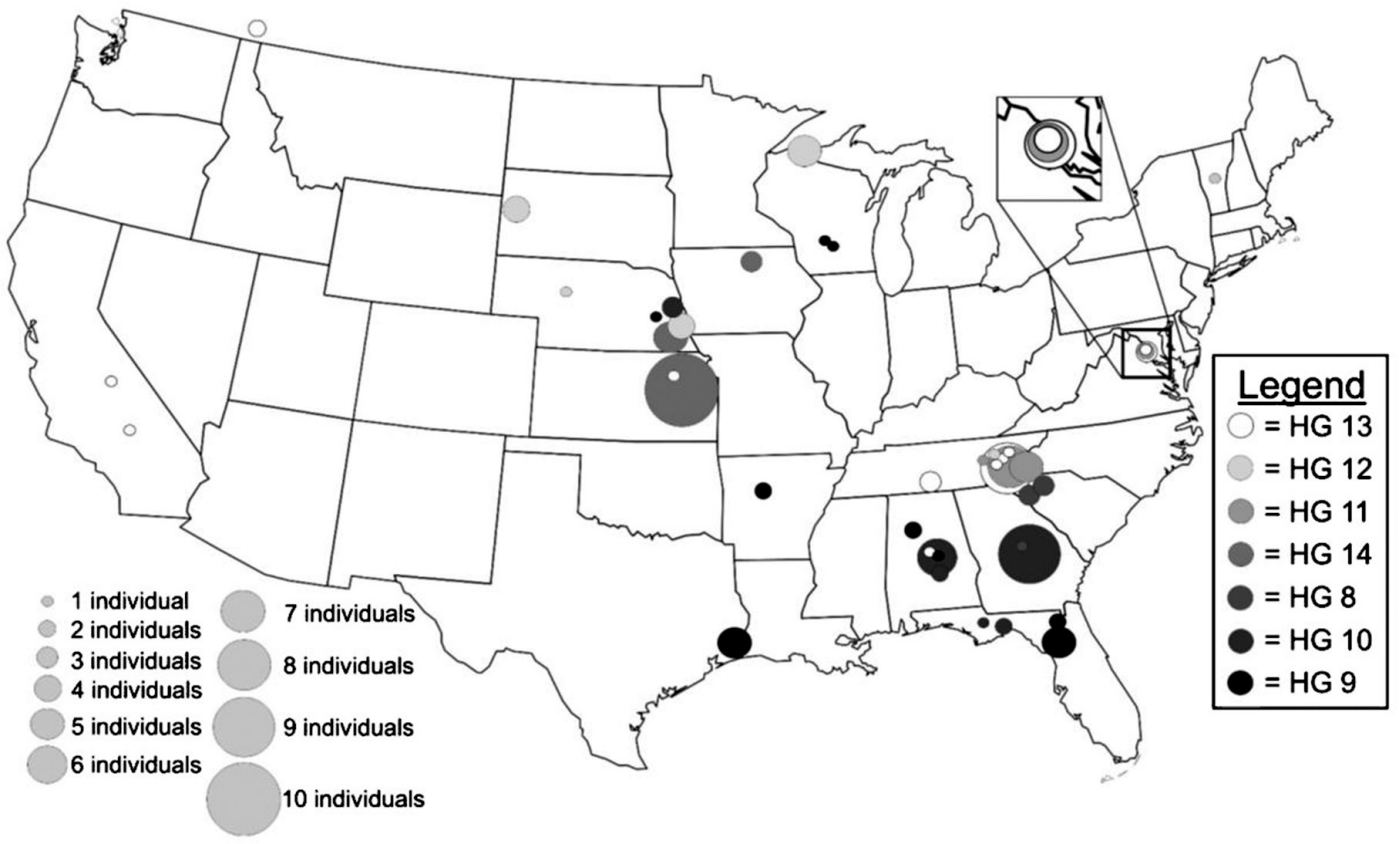
Distribution of *Mesocriconema xenoplax* HG per collection site within the United States. The color of the circle is associated with the HG number, and the size of the circle is associated with the number of individuals of each HG collected at each site. HG, haplotype group.

Nematodes included in HG 12 (*n* = 16) were collected from sites in Nebraska, South Dakota, Wisconsin, Tennessee, and Vermont, all from native plant communities (Fig. 2). Specimens comprising the sister clade to HG 12 and HG 13 (*n* = 28) were also predominantly collected from native plant communities, including forests in the Canadian province of British Columbia and five sites within Great Smoky Mountains National Park. Representatives of HG 13 were also collected from a vineyard in California near the type locality of *M. xenoplax* in the vicinity of Fresno. Both HG 12 and HG 13 are present in the Great Smoky Mountains and northern temperate forests, but not found south of the Appalachian Mountains. The sister group to HG 12 and 13 is HG 11. Like its sister clades, HG 11 was collected from multiple sites within Great Smoky Mountains National Park and northern Virginia, with one western sample obtained from a walnut farm in central California.

### Geographic distribution of HG 8 and 10

Two HG were predominantly comprised of specimens associated with peach orchards. HG 8 (*n* = 8) consisted of specimens exclusively collected from peach orchards in South Carolina, Georgia, and Alabama. There were only two haplotypes in HG 8, one representing all the specimens from South Carolina, and the other found in orchards in Georgia and Alabama. Nematodes in HG 10 (*n* = 23) were primarily collected from peach orchards in Georgia and Alabama. Three specimens from a nursery in Nebraska belonged to this group. Haplotype 10 also included specimens from two state parks in Florida. Specimens from Ichetucknee State Park in northern Florida shared a haplotype that was common in the Georgia peach orchards, whereas Torreya State Park located in the Florida panhandle contained a specimen differing by one nucleotide from haplotypes found in Alabama peach orchards.

### Geographic distribution of HG 14

HG 14 was the only HG not associated with North American forests. All specimens in this group were collected from remnant tallgrass prairie sites in Iowa, Kansas, and Nebraska. Precise host relationships have not been determined for this group, but collection sites were in the proximity of native woody shrubs that commonly invade prairie habitats, such as roughleaf dogwood (*Cornus drummondii* C.A. Mey.) and smooth sumac (*Rhus glabra* L.).

### Geographic distribution of HG 9

HG 9 has the greatest Hd, intragroup nucleotide diversity (Pi), and average number of nucleotide differences (k) (Table 5). It is also unique among the seven HG in that its haplotypes were collected from Florida to Texas from Gulf Coast forests. Six haplotypes were recorded in Big Thicket National Preserve in Texas, from collection sites that featured native pine trees. Two specimens recovered from native prairie sites in Wisconsin may suggest the role of the Mississippi River as a corridor for plant and animal dispersal.

## Population Structure

**Figure 3:**
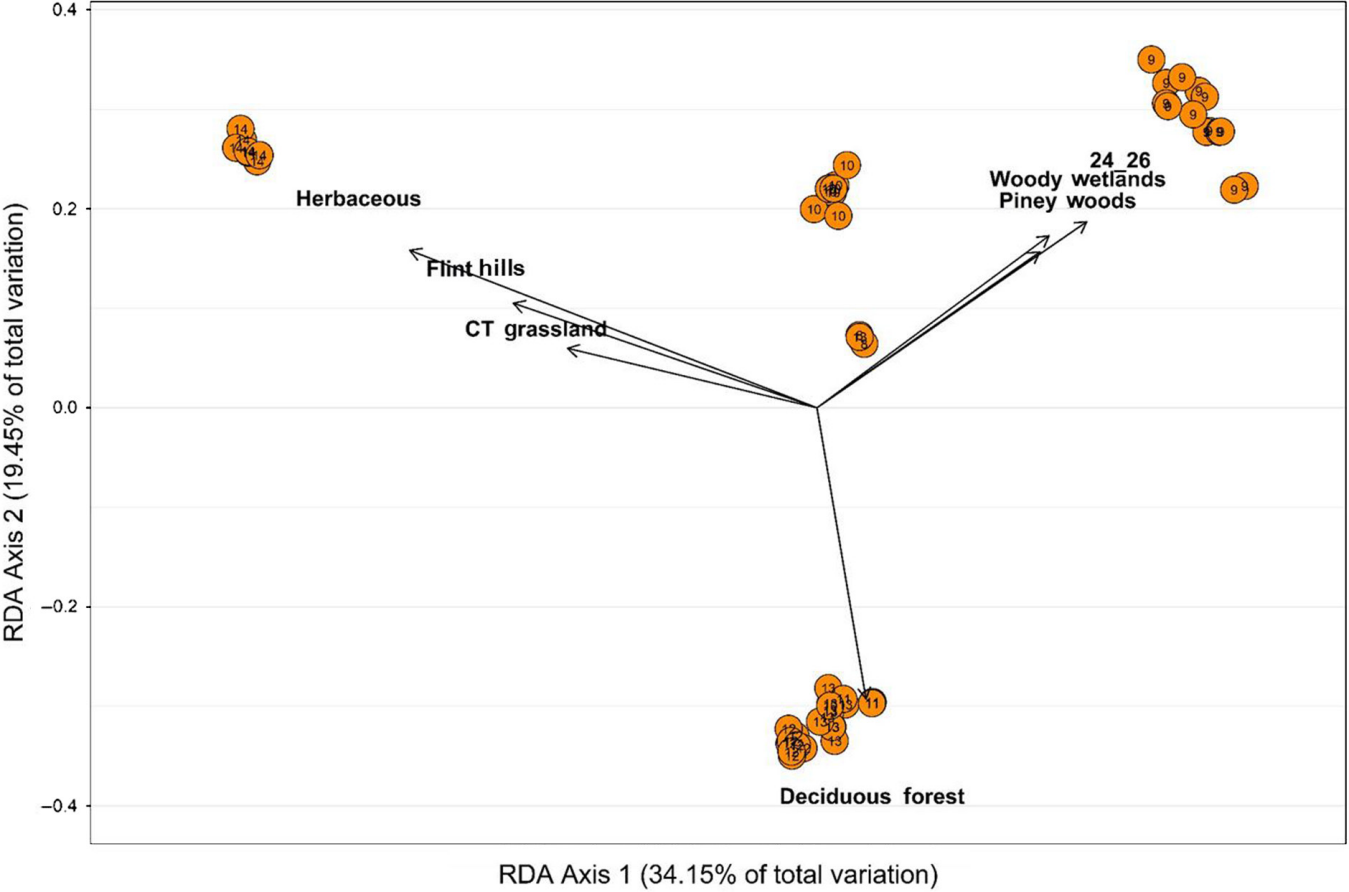
An ordination plot using dbRDA bi-plot showing the seven most influential explanatory environmental variables (arrows) overlain on the first two eigenvectors of the analysis of *Mesocriconema xenoplax* HG. The length of the arrows is directly proportional to the strength of the correlation between the explanatory variable and the genetic variation calculated between individuals within each HG. Circles represent HG in ordination space, with the corresponding HG number indicated in the respective circle. Each axis indicates the amount of total variation each respective axis accounts for. dbRDA, distance-based redundancy analysis; HG, haplotype group.

Results within the dbRDA analysis identified an autocorrelation (0.964) between minimum and maximum temperatures. Minimum temperature displayed less of the variation within the global model, a model that includes all possible variables; therefore, it was omitted from further analysis. Multivariate dbRDA analysis of the pairwise genetic distances and environmental metadata identified that the global model accounted for 35.7% (*F* = 3.990, *P* = 0.001) of the initial variation, with an adjusted *R*^2^ = 68.2% (*P* < 0.001). The function ordistep() identified the significant predictors of genetic variation from the global model. This reduced model included: land cover, ecoregion, and maximum temperature, accounting for 71.0% of the total variation. The reduced model was applied to the bi-plot analysis and explained a significant portion of the initial variation (*F* = 3.418, *P* = 0.001), with an adjusted *R*^2^ = 50.263 (*P* = 0.001; Fig. 3). The “herbaceous land cover” category was the strongest predictor, followed by maximum temperature, Flint Hills Tall Grassland ecoregion, deciduous forest and woody wetland land cover, and Piney Woods Forest and Central Tall Grassland ecoregions. The first axis accounted for 34.2% of the variation, and the second axis accounted for 19.5%. HG 14 associations included herbaceous land cover, the Flint Hill Tall Grassland ecoregion, and the Central Tall Grassland ecoregion. HG 11, 12, and 13 are associated with deciduous forest land cover. HG 9 is associated with a maximum temperature range of 23.88^o^C to 26.11^o^C, woody wetland land cover, and Piney Woods Forest ecoregion.

**Figure 4:**
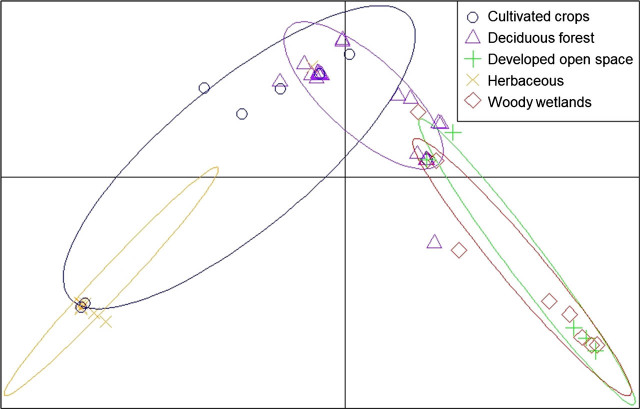
Scatterplots of discriminant analysis of principal components, calculated on the category of land cover that distinguishes the sequential data the most. Points represent observed individual *Mesocriconema xenoplax* nematodes identified at their respective land cover type, connected to the population centroids. The center of each component is represented as black grid lines. Land cover types with fewer than five individual nematodes were removed from the analysis to reduce the amount of noise.

**Figure 5A–E:**
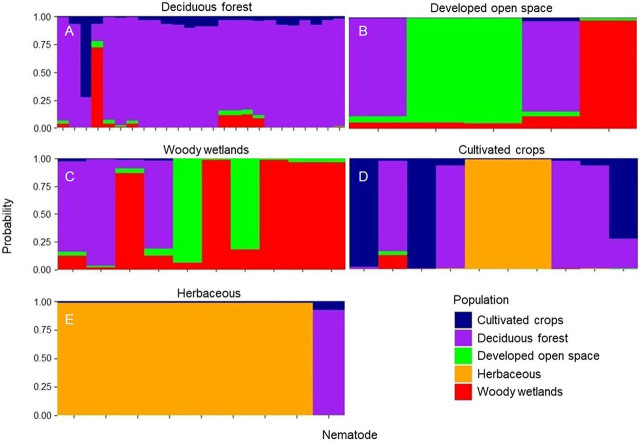
Stacked bar plot of the probability of each *Mesocriconema xenoplax* nematode to be reassigned to its respective population, defined according to land cover type; A. deciduous forest, B. developed open space, C. woody wetlands, D. cultivated crops, and E. herbaceous. Only those land cover types with ≥5 individuals were included in the analysis. The vertical axis is the probability (y-axis) of each individual (x-axis) being reassigned to its respective population, and the width of the bars depends on population size.

Based on the dbRDA results, land cover was influential for the separation of three HG and was the strongest predictor variable. Therefore, land cover was analyzed further using the discriminant analysis of principal components. The first seven principal components were analyzed, saving four discriminant functions with 92.9% of the variation accounted for. The first discriminant axis represented 11.0% of the variation, and the second axis represented 4.2% (Fig. 4). In general, individuals were clustered loosely near herbaceous land cover, which was the most isolated variable on the bi-plot. The pairwise AMOVA indicated differentiation with significant differences between nematodes from herbaceous and woody wetlands (*P* ≤ 0.01; Table 7). Visualization of individual membership to each of the land cover categories indicated an average reassignment of 52.4% in the category of deciduous forest (min = 17.9%, max = 80.2%, SD = 30.3%; Fig. 5A–E), followed by an average reassignment of 35.4% for woody wetlands (min = 1.5%, max = 67.6%, SD = 30.3%), 10.3% for cultivated crops (min = 0.8%, max = 24.6%, SD = 10.1%), 0.9% for developed open space (min < 0.1%, max = 2.6%, SD = 1.1%), and 0.9% for herbaceous land cover (min < 0.1%, max = 3.6%, SD = 1.0%).

**Table 7:** Population comparison defined by four categories of land use associated with the morphospecies *Mesocriconema xenoplax* in a pairwise AMOVA.

	**Number of Individuals**	**Deciduous forest**	**Developed open space**	**Woody wetlands**	**Cultivated crops**	**Herbaceous**
Deciduous forest	25	–	0.010	<0.001	0.011	<0.001
Developed open space	5	0.263	–	0.92	0.168	0.001
Woody wetlands	10	0.375	–0.114	–	0.027	<0.001
Cultivated crops	10	0.118	0.087	0.217	–	0.009
Herbaceous	9	0.654	0.725	0.757	0.330	–

The lower triangle displays the statistic *ϕ*, and the value with the upper triangle displays the statistical significance of the *ϕ* statistic. Land cover types with fewer than five individual nematodes were removed from the analysis. AMOVA, analysis of molecular variance.

### Phenotypic variation

**Figure 6:**
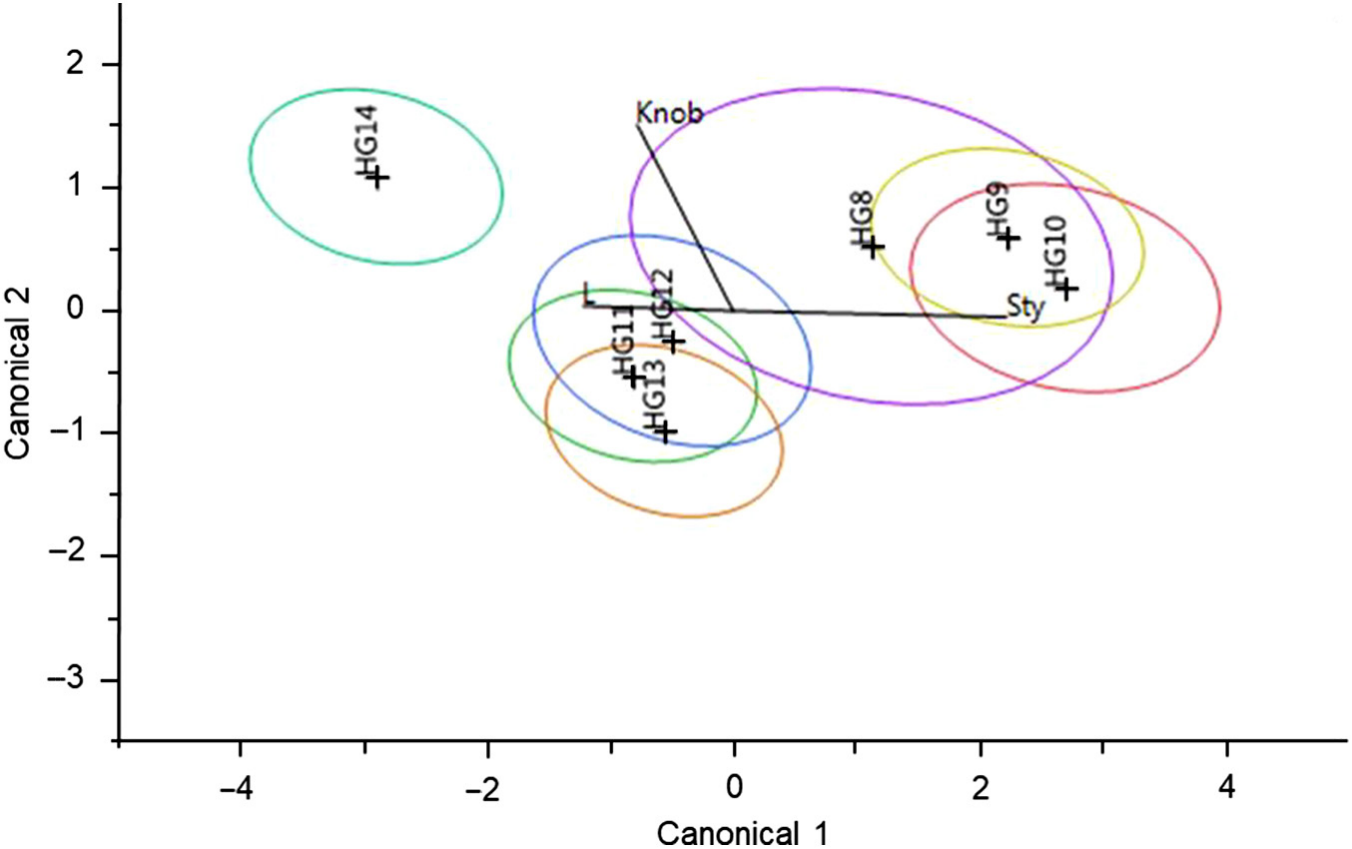
Canonical plot of the seven *Mesocriconema xenoplax* HG are indicated by color and significant morphological features: knob width (knob), total body length (L), and stylet length (Sty). Rays are scaled in the canonical plots to 1.5. The larger ellipses denote 50% of the observations, assuming normality, respective to size, for the mean canonical variables. The smaller ellipses represent a 95% confidence ellipsoid for the means on the canonical variable. HG, haplotype group.

Morphological analysis of each HG in the discriminant function analysis identified that stylet length, total body length, and stylet knob width are the strongest distinguishing features among the seven HG as defined a priori (Fig. 6), and stylet length to be the strongest distinguishing feature. The analysis correctly classified 61 out of 87 (70.1%) of the sequences belonging to their respective groups (misclassifying 26). Canonical discriminant function for the first dimension indicated that stylet length and total body length accounted for most of the variation between groups (85.8% variation; *Wilk’s λ* = 0.189, *F_18_* = 13.059, *P* < 0.001). The eigenvector for stylet length extended longer than the eigenvectors for knob width, which were positioned at an angle close to 90^o^C, suggesting little to no relationship between the two morphological features in their ability to differentiate between groups. Canonical discriminant function for the second dimension indicated that stylet length and knob width are inversely related and account for less variation than the first dimension (13.4%; *Wilk’s* λ = 0.606, *F_10_* = 4.426, *P* < 0.001). The third dimension of canonical correlations was not significant. Body length and knob width were slightly related as they are on a similar angle, with less than a 90^o^ angle and knob width having a slightly longer ray. HG 9 and 10, and HG 11, 12, and 13 formed two well-separated clusters. HG 8 overlapped both of these clusters. HG 14 was isolated, with no overlap with any other HG. HG 14 had the highest mean body length (641.2 μm) and knob width (13.8 μm), and the smallest stylet length (70.4 μm). HG 9 had the second largest mean body length (634.8 μm), knob width (13.5 μm), and stylet length (87.6 μm). HG 13 had the smallest body length (average 578 μm) and knob width (11.5 μm), and the second-smallest stylet length (72.3 μm).

## Discussion

In this study, we sought to investigate the ecological and morphological groupings within *M. xenoplax* using multiple approaches to determine if the resulting groupings support one large, widespread, generalist species or multiple, genetically distinct, sub-populations, occupying specific niches. To test this, we focused on 132 samples that originated from a broad range of environments, ecosystems, habitats, and land cover patterns throughout the USA and Canada. Using COI DNA Sanger sequencing of individual specimens, we applied an integrated approach to clarify haplotype groupings for *M. xenoplax* using three categories of genetic analyses: phylogenetic, species delimitation, and population structure. In this study, phylogenetic and species delimitation analyses provided ample support for seven independently evolving lineages (Powers *et al*., 2014). Also in this study, population structure analyses identified differentiation for three populations by environmental variables, land cover, and ecoregion (Porazinska *et al*., 2003; Darby *et al*., 2011, Duyck *et al*., 2012; Tsiafouli *et al*., 2017). Morphological analysis identified distinction for groups based on total body length, stylet length, and stylet knob width.

Support for HG 9 and 14, and for the combination of HG 11, 12, and 13, were recognized based on environmental variables. HG 14 stood out as a group found only in native prairies and associated with midwestern prairie ecoregions and herbaceous land cover type. This distribution pattern has previously been observed in other criconematid nematode species (Powers *et al*., 2016; Olson *et al*., 2017). Of the seven land cover types, HG 11, 12, and 13 were distinguished by an association with deciduous forests, particularly within the GSMNP region and expanding northward. Members of these groups were not observed south of GSMNP. The AMOVA indicated that only deciduous forest and herbaceous land cover types were significantly different from all other land cover types. Morphological measurements distinguished HG 14, HG 9 and 10, and HG 11, 12, and 13. Future studies could explore the relationship between morphology and environment further, focusing on host–parasite relationships, particularly the degree to which species of plant parasites are generalists or host specialists. We were unable to find consistent support in our measurement of isolation by distance within HG, due to the lack of sufficient sample size (Castellano and Balletto, 2002; Lycett *et al*., 2011).

Our study was the first to analyze the population structure of *M. xenoplax*, combining genetic variation with environmental and morphological variations to identify predictive patterns of HG distribution and to highlight possible new species boundaries within *M. xenoplax*. Based on these results, we believe that there is sufficient support for the recognition of one new species, comprised of HG 14, based on genetic, morphological, and ecological distinction. Other haplotype groupings might be supported as separate species, but we feel that additional evidence is necessary to fully assess species status. We also believe that the composition of the plant community at the scale of land cover and ecoregion is a potential indicator for subdivisions with *M. xenoplax* as observed in our population structure analysis. These conclusions parallel similar studies that recognize species boundaries of an organism by combining biological, physiological, and genetic data for the establishment of species boundaries within otherwise cryptic species (Cairns *et al*., 1993; Porazinska *et al*., 1999; Barbercheck *et al*., 2009; Shao and Tu, 2012). However, it was not the objective of this paper to describe new species in *Mesocriconema xenoplax* and await the clarification of host relationship analyses.

A surprising result of this study is the association of specific HG with agroecosystems, which has the potential to influence management strategies for nematodes feeding on agroeconomic hosts. In particular, the results have implications for the process of breeding for resistance in agronomic hosts, as different genetic populations may exhibit different physiological responses. Collectively, our study provides multiple lines of evidence that the morphospecies named *M. xenoplax* contains separately evolving meta-populations that display ecological differentiation. In a broader sense, these results allow us to better understand the interactions that plant parasitic nematodes have historically maintained within specific environments and hosts, shedding light on how these associations may serve to predict nematode distributions in a changing environment.
